# Real-world use of semaglutide in patients with type 2 diabetes and end-stage renal disease: a multicenter retrospective cohort study

**DOI:** 10.3389/fendo.2026.1805015

**Published:** 2026-03-26

**Authors:** Omar Alkhezi, Lama Alfehaid, Amal Alanezi, Monirah Alotaibi, Omar Alshaya

**Affiliations:** 1 Department of Pharmacy Practice, College of Pharmacy, Qassim University, Al-Qassim Buraydah, Qassim, Saudi Arabia; 2 Pharmacy Practice Department, College of Pharmacy, King Saud bin Abdulaziz University for Health Sciences, Riyadh, Saudi Arabia; 3 Department of Pharmaceutical Care Services, King Abdulaziz Medical City, Ministry of National Guard-Health Affairs, Riyadh, Saudi Arabia; 4 King Abdullah International Medical Research Center (KAIMRC), Ministry of National Guard Health Affairs (MNGHA), Riyadh, Saudi Arabia

**Keywords:** cardiovascular outcomes, end-stage renal disease, chronic kidney disease, glucagon-like peptide-1 receptor agonists, hemodialysis, real-world evidence, semaglutide, type 2 diabetes mellitus

## Abstract

**Background:**

Semaglutide and other Glucagon-like peptide-1 receptor agonists (GLP-1 RAs) have demonstrated cardiovascular and renal benefits in patients with type 2 diabetes mellitus (T2DM); however, individuals with end-stage renal disease (ESRD) have been systematically excluded from landmark outcome trials. Consequently, real-world data evaluating the safety and effectiveness of GLP-1 RAs in this high-risk population remain limited.

**Methods:**

This multicenter retrospective cohort study evaluated adult patients with T2DM and ESRD, including those receiving maintenance hemodialysis, who were prescribed injectable semaglutide between January 2016 and July 2025 at tertiary care centers in Saudi Arabia. The primary efficacy outcomes were changes in glycemic control and insulin requirements following initiation of injectable semaglutide. Secondary outcomes included changes in body weight. Safety outcomes comprised acute kidney injury (AKI), severe hypoglycemia, treatment discontinuation due to adverse events, expanded major adverse cardiovascular events (MACE), and all-cause mortality.

**Results:**

Seventeen patients were included, with a median follow-up of 1,187 days (IQR 602–1,442); 58.8% were receiving hemodialysis. Mean HbA1c decreased from 9.06 ± 1.69% to 8.75 ± 2.48% (−0.31 ± 2.57%; p = 0.630), with insulin dose reductions observed among the subset of patients with available documentation. Among 16 patients with paired weight measurements, mean body weight decreased by −12.63 ± 24.03 kg (p = 0.074). No cardiovascular deaths, nonfatal myocardial infarctions, or nonfatal strokes were observed. Three expanded MACE events, all hospitalizations for heart failure, occurred during follow-up. AKI occurred in 57.1% of non-dialysis patients, and severe hypoglycemia was reported in two patients (11.8%).

**Conclusions:**

In real-world practice, injectable semaglutide was associated with descriptive changes in metabolic parameters in patients with ESRD, including those on hemodialysis. Larger prospective studies are needed to better define the role of GLP-1 RAs in this underrepresented population.

## Introduction

Diabetes mellitus (DM) remains a major global public health challenge and is the leading cause of chronic kidney disease (CKD) and end-stage renal disease (ESRD) worldwide. The global prevalence of diabetes continues to rise, with projections indicating a substantial increase in disease burden over the coming decades (1, 2). Approximately 30–40% of individuals with type 2 diabetes mellitus (T2DM) develop diabetic kidney disease (DKD), which is the predominant cause of ESRD and a major contributor to cardiovascular morbidity and mortality (2, 3).

Patients with T2DM and ESRD represent a particularly high-risk population characterized by advanced metabolic dysfunction, a high burden of cardiovascular disease, and extensive polypharmacy (4). Glycemic management in advanced CKD and ESRD is challenging due to altered drug pharmacokinetics, reduced renal clearance, and increased susceptibility to hypoglycemia. Many conventional glucose-lowering therapies require dose adjustment or are contraindicated in severe renal impairment, leaving limited therapeutic options. Identifying treatments that are both effective and safe in advanced kidney disease, therefore, remains a critical unmet need. In addition, weight reduction is clinically meaningful in patients with ESRD, as it may improve cardiovascular risk profiles, dialysis adequacy, and eligibility for kidney transplantation.

Glucagon-like peptide-1 receptor agonists (GLP-1 RAs) improve glycemic control by increasing glucose-dependent insulin secretion, suppressing glucagon release, delaying gastric emptying, and inducing weight loss. Beyond their metabolic effects, GLP-1 RAs have demonstrated cardiovascular and renal benefits in large outcome trials, including reductions in major adverse cardiovascular events (MACE) and attenuation of albuminuria progression (5–17). However, patients with ESRD and those receiving maintenance dialysis have been systematically excluded from these pivotal trials. As summarized in **Table 1**, renal eligibility criteria across landmark GLP-1 RA cardiovascular and kidney outcome trials consistently excluded dialysis-dependent patients, resulting in a persistent lack of randomized evidence in this high-risk population.

**Table 1:** Landmark GLP-1 receptor agonist trials and renal eligibility.

Trial/setting	GLP-1 RA	Population	Kidney inclusion/exclusion (eGFR)	Dialysis allowed	GFR range studied	Major clinical and renal findings
LEADER (5)	Liraglutide 1.8 mg	T2DM + high CV risk	Included CKD, including severe renal impairment (eGFR <30), but excluded dialysis	No	Included <30 (non-dialysis)	↓ major adverse CV events; ↓ composite renal outcome, driven primarily by reduced progression of albuminuria
SUSTAIN-6 (6)	Semaglutide 0.5–1.0 mg s.c.	T2DM + high CV risk	Excluded chronic dialysis; included severe CKD (randomization stratified by eGFR ≤30 vs >30)	No	Included severe CKD stratum (non-dialysis)	↓ CV events (noninferiority); ↓ new or worsening nephropathy in prespecified renal analyses
PIONEER-6 (7)	Oral semaglutide 14 mg	T2DM + high CV risk	Excluded eGFR <30 and excluded dialysis	No	≥30	CV safety demonstrated; favorable kidney function trends observed in *post hoc* analyses
REWIND (8)	Dulaglutide 1.5 mg weekly	Broad T2DM population	Excluded stage 5 CKD (eGFR <15) or dialysis	No	≥15	↓ major adverse CV events; ↓ composite renal outcome, largely driven by reduced macroalbuminuria
AWARD-7 (9)	Dulaglutide 0.75/1.5 mg	T2DM + CKD stage 3–4	Included eGFR 15–<60; excluded dialysis	No	15–<60	Comparable glycemic control vs insulin glargine; slower eGFR decline and reduced albuminuria
FLOW (10)	Semaglutide 1 mg weekly	T2DM + albuminuria CKD	Included eGFR 25–75; excluded dialysis	No	25–75	↓ major kidney events (primary outcome); ↓ risk of sustained eGFR decline and kidney failure
STEP Program (11)	Semaglutide 2.4 mg	Obesity ± T2DM	Severe renal impairment/ESRD generally excluded; cutoffs varied by trial	No (in general)	Mostly preserved kidney function	Significant weight loss; kidney outcomes not primary and not systematically assessed
SELECT (12)	Semaglutide 2.4 mg	Obesity + CVD, no diabetes	ESRD/hemodialysis/peritoneal dialysis excluded; severe CKD uncommon	No	Mostly ≥30	↓ major adverse CV events; ↓ kidney composite outcome with improved eGFR slope in prespecified analyses
SCALE Program (13)	Liraglutide 3.0 mg	Obesity ± prediabetes	Severe renal impairment/ESRD excluded; cutoffs varied by trial	No	Generally non-ESRD	Significant weight loss; no major renal safety signal observed
HARMONY Outcomes (14)	Albiglutide 30–50 mg weekly	T2DM + established CVD	Excluded eGFR <30; excluded dialysis	No	≥30	↓ major adverse CV events; renal outcomes not primary, with no major renal safety signal
EXSCEL (15)	Exenatide once weekly 2 mg	Broad T2DM population	Excluded eGFR <30 mL/min/1.73 m²	No	≥30	CV noninferiority demonstrated; renal outcomes not primary and no major renal safety signal reported
ELIXA (16)	Lixisenatide 20 µg daily	T2DM + recent acute coronary syndrome	Excluded eGFR <30 mL/min/1.73 m²	No	≥30	CV safety demonstrated; renal outcomes not primary and no major renal safety signal reported
AMPLITUDE-O (17)	Efpeglenatide weekly	T2DM + CVD and/or CKD	Excluded eGFR <15; excluded dialysis	No	≥15	↓ major adverse CV events; ↓ composite kidney outcome including sustained eGFR decline

Renal eligibility criteria are reported as specified in the primary trial publications and protocols; most cardiovascular outcome trials excluded patients with end-stage renal disease (ESRD) or those receiving maintenance dialysis.

Dialysis Allowed refers to the inclusion of patients receiving chronic hemodialysis or peritoneal dialysis at baseline; none of the listed trials enrolled patients receiving maintenance dialysis.

Major clinical and renal findings reflect prespecified primary or key secondary outcomes where applicable; in trials without prespecified kidney endpoints, renal findings are derived from secondary, *post hoc*, or safety analyses.

In cardiovascular outcome trials (CVOTs), renal outcomes were generally exploratory and frequently driven by changes in albuminuria rather than hard kidney endpoints.

FLOW was the only trial listed with a primary kidney outcome, specifically designed to assess the effect of a GLP-1 receptor agonist on major kidney events.

Obesity trials (STEP and SCALE programs) were not designed to evaluate renal outcomes; kidney function assessments in these studies primarily served safety monitoring purposes.

Reported eGFR thresholds refer to estimated glomerular filtration rate calculated using trial-specified equations and may differ slightly across studies.

CV safety indicates noninferiority for major adverse cardiovascular events when superiority was not demonstrated.

↓ Means lower

Emerging observational studies and small interventional trials suggest that GLP-1 RAs may be used in patients with advanced CKD with preserved glucose-lowering efficacy and a relatively low intrinsic risk of hypoglycemia compared with insulin and sulfonylureas (18–23). A systematic review and meta-analysis including patients with reduced kidney function (eGFR <60 mL/min/1.73 m²) demonstrated improvements in cardiovascular and kidney outcomes and reductions in all-cause mortality without increased risk of severe hypoglycemia, pancreatitis, or pancreatic cancer (24). Real-world data have also suggested potential cardiovascular and renal benefits of GLP-1 RAs in advanced CKD compared with other glucose-lowering therapies (25). Nevertheless, patients with ESRD remain markedly underrepresented in these analyses.

Semaglutide has recently received expanded regulatory approval for use in patients with T2DM and CKD, reflecting growing confidence in its renal safety profile and potential kidney-protective benefits (26, 27). However, high-quality evidence specific to patients with ESRD or those receiving dialysis remains limited, and uncertainty persists regarding safety, tolerability, and real-world effectiveness in this population.

In Saudi Arabia, T2DM prevalence is among the highest worldwide and is accompanied by high rates of obesity, cardiovascular disease, and CKD (28). Although GLP-1 receptor agonists are increasingly used in clinical practice, data evaluating their safety and effectiveness in patients with ESRD are scarce. Given regional differences in patient characteristics and healthcare delivery systems, locally generated evidence is essential.

Therefore, this multicenter retrospective study aimed to evaluate the real-world safety and effectiveness of semaglutide in patients with ESRD receiving care at Saudi tertiary healthcare centers, with a focus on glycemic outcomes, body weight changes, cardiovascular events, renal safety, and treatment tolerability.

## Methods

### Study design and setting

This was a multicenter retrospective cohort study conducted across tertiary care hospitals in Saudi Arabia, including Ministry of National Guard Health Affairs (MNGHA) medical cities in Riyadh, Jeddah, and Al-Ahsa. Data were obtained from electronic medical record systems, *BESTCARE*, routinely used for clinical care.

### Study population

Adult patients (≥18 years) with T2DM and ESRD who were prescribed a GLP-1 RA between January 1, 2016, and July 17, 2025, were eligible for inclusion. ESRD was eGFR <15 mL/min/1.73 m² documented on at least two measurements ≥30 days apart and/or receipt of maintenance dialysis at the time of or following GLP-1 RA initiation. Eligible patients were required to have received at least one prescription of a GLP-1 RA for the management of diabetes or obesity during the study period. Patients were included regardless of dialysis modality to reflect real-world prescribing practices in tertiary care centers. Patients with acute kidney injury (AKI) or unstable renal function at baseline were excluded. Individuals receiving substantial components of their medical care outside the participating institutions were also excluded to ensure complete capture of laboratory, medication, and outcome data. No patients with advanced CKD stage 4 (eGFR 15–29 mL/min/1.73 m²) were included. The final cohort consisted exclusively of patients meeting ESRD criteria as defined above.

### Data collection

Demographic characteristics, anthropometric measures, comorbid conditions, duration of diabetes and renal disease, dialysis status and modality, and concomitant glucose-lowering therapies were extracted from the electronic medical record system. Body weight values were obtained from routine clinical documentation and were not standardized in relation to dialysis sessions (e.g., pre- versus post-dialysis measurements), reflecting real-world clinical practice. Laboratory variables included hemoglobin A1c, fasting and postprandial blood glucose levels, and available renal parameters. Cardiovascular history and clinical events were identified through diagnostic codes and clinician documentation within the medical record. Semaglutide exposure was characterized using prescription-level data obtained from the institutional electronic medication database. Extracted variables included prescription dates and prescribed weekly doses, which were used to determine initial dose, maximum dose achieved, dose escalation patterns, and treatment duration based on prescription span (first to last recorded prescription). Patients without retrievable semaglutide prescription records within the electronic medication database were excluded from dose-level exposure analyses but remained included in overall clinical and safety outcome analyses to preserve cohort integrity.

### Outcomes

The primary efficacy outcome was the change in glycemic control following initiation of GLP-1 RA therapy. Glycemic control was assessed by changes in hemoglobin A1c and clinically meaningful modifications in diabetes management, including reductions or discontinuations of basal or bolus insulin therapy. Changes in insulin therapy were included as pragmatic surrogate markers of improved glycemic stability in a population at high risk for hypoglycemia and treatment-related adverse events.

Secondary efficacy outcomes included change in body weight following initiation of GLP-1 RA therapy.

Safety outcomes included the occurrence of AKI, severe hypoglycemic events requiring medical attention, and discontinuation of GLP-1 RA therapy due to adverse effects, including pancreatitis and gallstones (cholelithiasis).

Pancreatitis and gallstones were identified based on clinician-documented diagnoses within the electronic medical record. These events were captured through chart documentation and diagnostic coding during follow-up. Structured laboratory parameters (e.g., lipase or amylase levels) and imaging data were not systematically extracted for independent adjudication.

Discontinuation events were initially identified through structured prescription records within the electronic medication database and subsequently adjudicated through review of clinician documentation and diagnostic coding in the electronic medical record. In cases of discrepancy between structured prescription fields and chart documentation, clinician-documented diagnoses were considered the primary source for classification of discontinuation cause.

Gastrointestinal adverse events were not collected as predefined outcomes unless they resulted in documented treatment discontinuation; therefore, minor or transient gastrointestinal symptoms may be underrepresented.

AKI was defined according to the Kidney Disease: Improving Global Outcomes (KDIGO) consensus serum creatinine criteria as either an increase in serum creatinine of ≥0.3 mg/dL (≥26.5 μmol/L) within 48 hours or an increase to ≥1.5 times baseline within the preceding 7 days (29). Urine output criteria (<0.5 mL/kg/h for at least 6 hours) were not applied due to inconsistent documentation in the electronic medical record. AKI analyses were restricted to patients not receiving maintenance dialysis, as creatinine-based KDIGO definitions are not standardized in dialysis-dependent ESRD. Recurrent AKI was defined as more than one distinct AKI episode during follow-up.

Cardiovascular outcomes were evaluated using a prespecified composite endpoint. Expanded major adverse cardiovascular events (MACE) were defined as a composite of cardiovascular death, nonfatal myocardial infarction, nonfatal ischemic stroke, and hospitalization for heart failure. Each component was identified through clinician-documented diagnoses and corresponding diagnostic codes within the electronic medical record. Each patient contributed only the first occurrence of any component to the composite endpoint; no patient experienced more than one expanded MACE event.

Coronary revascularization and hospitalization for unstable angina were analyzed and reported separately as individual cardiovascular outcomes and were not included in the expanded MACE composite. All-cause mortality was assessed as an independent outcome. Cause-specific mortality (cardiovascular versus non-cardiovascular) was not independently adjudicated due to limitations in structured documentation within the electronic medical record system; therefore, deaths were classified descriptively based on available documentation.

### Follow-up

Patients were followed from the index date (the date of GLP-1 receptor agonist prescription) until death, loss to follow-up, or the end of the study period (December 1, 2025), whichever occurred first. Follow-up was not standardized to prespecified assessment windows; all outcomes reflect the most recent available clinical measurements documented in the electronic medical record system during routine care.

### Statistical analysis

Changes in continuous outcomes from baseline, including hemoglobin A1c and body weight, were evaluated using paired statistical methods. Given the small sample size and the anticipated non-normal distribution of within-patient changes in individuals with ESRD, the Wilcoxon signed-rank test was prespecified as the primary analytic approach. Paired t-tests were performed as sensitivity analyses where appropriate to assess the consistency of results. Reported p-values correspond to the Wilcoxon signed-rank test unless otherwise specified.

Continuous variables are presented as mean ± standard deviation (SD) or median (interquartile range [IQR]), as appropriate. For key continuous outcomes, 95% confidence intervals were estimated using bootstrap resampling due to the limited sample size.

Binary clinical outcomes, including acute kidney injury, severe hypoglycemia, adverse events leading to treatment discontinuation, and cardiovascular events, were summarized descriptively without formal hypothesis testing, given the absence of a comparator group and limited statistical power. All statistical tests were two-sided, and a p-value <0.05 was considered statistically significant where applicable.

### Ethical consideration

The study was conducted in accordance with institutional and national ethical standards. Patient confidentiality was maintained through the de-identification of data, and all records were stored securely with restricted access. Institutional review board approval was obtained (NRR25/096/5, approval No. 00000169925) with a waiver of informed consent due to the study’s retrospective nature.

## Results

**Figure 1:**
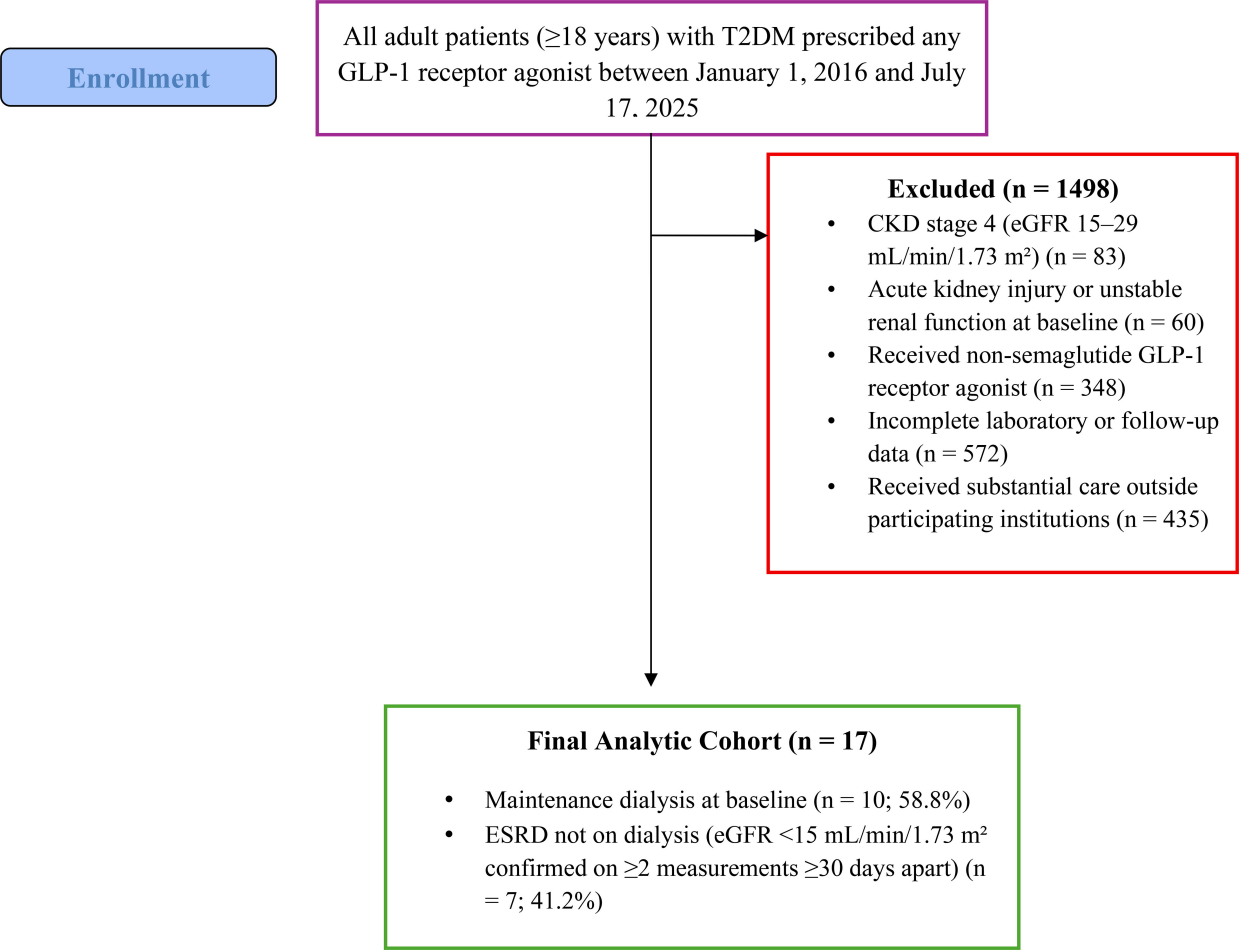
Flow diagram of patient screening, exclusions, and final analytic cohort.

Between January 1, 2016, and July 17, 2025, a total of 1,515 adult patients with T2DM who were prescribed a GLP-1 receptor agonist were screened. After applying predefined inclusion and exclusion criteria, 17 patients with confirmed ESRD who received injectable semaglutide were included in the final analytic cohort. The study selection process is illustrated in **Figure 1**.

### Baseline characteristics

Seventeen patients with T2DM and confirmed ESRD were included. Of these, 10 (58.8%) were receiving maintenance dialysis at baseline, while 7 (41.2%) had ESRD not yet requiring dialysis, defined as eGFR <15 mL/min/1.73 m² confirmed on two measurements ≥30 days apart.

The mean age was 50.5 ± 18.8 years, and 13 patients (76.5%) were female. Mean baseline hemoglobin A1c was 9.06 ± 1.69%. Mean baseline body weight was 98.6 ± 23.3 kg, mean BMI was 38.0 ± 7.2 kg/m², and mean eGFR was 16.5 ± 7.2 mL/min/1.73 m².

Hypertension was present in all patients (100%). Six patients (35.3%) had a history of ASCVD, and seven (41.2%) had heart failure. Baseline characteristics are summarized in **Table 2**.

**Table 2:** Baseline characteristics of the study population (n=17).

Characteristic	Overall
Demographics	
Age, years (mean ± SD)	50.5 ± 18.8
Female sex, n (%)	13 (76.5)
Receiving dialysis, n (%)	10 (58.8)
Follow-up duration, days (median [IQR])	1187 [602–1442]
GLP-1 RA agent	Semaglutide injection, 17 (100)
Baseline laboratory and anthropometric measures	
Baseline HbA1c, % (mean ± SD)	9.06 ± 1.69
Baseline weight, kg (mean ± SD)	98.6 ± 23.3
Baseline BMI, kg/m² (mean ± SD)	38.0 ± 7.2
Baseline eGFR, mL/min/1.73 m² (mean ± SD)	16.5 ± 7.2
Comorbidities	
Hypertension, n (%)	17 (100.0)
ASCVD history, n (%)	6 (35.3)
– Acute coronary syndrome	1 (16.7% of ASCVD)
– Stable ischemic heart disease	2 (33.3% of ASCVD)
– Cerebrovascular disease	3 (50.0% of ASCVD)
Heart failure, n (%)	7 (41.2)
– HFmrEF	1 (14.3% of HF)
– HFpEF	6 (85.7% of HF)
Diabetic retinopathy, n (%)	11 (64.7)
Diabetic neuropathy, n (%)	1 (5.9)
Diabetic nephropathy, n (%)	8 (47.1)
Baseline diabetes medications	
Insulin (any), n (%)	16 (94.1)
– Basal insulin	14 (82.4)
– Bolus/mixed insulin	12 (70.6)
Metformin	0 (0.0)
Sulfonylurea	1 (5.9)
DPP-4 inhibitor	1 (5.9)
SGLT2 inhibitor	4 (23.5)
Baseline cardiovascular medications	
Beta blockers	10 (58.8)
ARNI	0 (0.0)
ACE inhibitors	1 (5.9)
ARBs	9 (52.9)
SGLT2 inhibitors	4 (23.5)
MRA	0 (0.0)

The median follow-up duration was 1,187 days (IQR 602–1,442).

### Semaglutide exposure

Prescription-level semaglutide data were available for 14 of 17 patients (82.4%). Three patients did not have retrievable prescription records within the electronic medication database and were therefore excluded from dose-level exposure analyses but remained included in clinical and safety analyses.

Among patients with available prescription data (n = 14), semaglutide was most commonly initiated at 0.25 mg or 0.5 mg weekly; 28.6% started directly at 1.0 mg weekly. Dose escalation during follow-up was documented in 8 patients (57.1%). The maximum achieved dose was 0.5 mg weekly in 14.3% and 1.0 mg weekly in 85.7% of patients.

The median time to first recorded dose escalation was 0 days (IQR 0–0), reflecting that dose adjustments were often documented at the time of the initial electronic prescription entry.

The median prescription span was 482 days (IQR 161–925), corresponding to 15.9 months (IQR 5.3–30.4 months).

Four patients (28.6% of those with exposure data) discontinued semaglutide due to safety events. The median time to discontinuation was 334.5 days (IQR 221–541 days). Based on clinician-documented diagnoses and diagnostic coding within the electronic medical record, one discontinuation was attributed to pancreatitis and three to gallstones (cholelithiasis). Discontinuation timing was identified through structured prescription records and subsequently adjudicated by chart review, with clinician documentation serving as the primary source for classification in cases of discrepancy. Exposure characteristics are summarized in **Table 3**.

**Table 3:** Semaglutide exposure characteristics among the study cohort.

Exposure variable	Overall (n = 14)
Starting dose: 0.25 mg weekly	4 (28.6%)
Starting dose: 0.5 mg weekly	6 (42.9%)
Starting dose: 1 mg weekly	4 (28.6%)
Maximum dose achieved: 0.5 mg weekly	2 (14.3%)
Maximum dose achieved: 1 mg weekly	12 (85.7%)
Any dose escalation (titration) documented	8 (57.1%)
Time to first dose escalation, days	0 (0–0)
Time on semaglutide (prescription span), days	482 (161–925)
Time on semaglutide (prescription span), months	15.9 (5.3–30.4)
Discontinuation due to safety issues	4 (28.6%)
Time to discontinuation, days	334.5 (221–541)
Discontinuation due to pancreatitis	1 (7.1%)
Discontinuation due to gallstones (cholelithiasis)	3 (21.4%)

Starting and maximum doses were derived from structured electronic prescription records. Time on semaglutide represents the interval between the first and last recorded semaglutide prescriptions for each patient (prescription span) and does not confirm adherence. Time to titration is defined as the number of days from the first prescription to the first recorded higher dose. Discontinuation timing was identified through prescription records, and causes for discontinuation were adjudicated based on clinician documentation and diagnostic coding within the electronic medical record. Values for continuous variables are presented as median (IQR).

### Glycemic control

Mean hemoglobin A1c decreased from 9.06 ± 1.69% at baseline to 8.75 ± 2.48% at the most recent available assessment, corresponding to a mean change of −0.31 ± 2.57% (p = 0.630). The estimated mean difference was −0.31% (95% bootstrap CI −1.46% to 0.91%), indicating substantial variability in glycemic response.

The median time from initiation of semaglutide to the most recent HbA1c measurement was 928 days (IQR 216–1,343; range 34–1,529). Most patients had prolonged follow-up: 76.5% had ≥6 months and 64.7% had ≥12 months of HbA1c follow-up. These findings represent descriptive longitudinal observations rather than standardized timepoint efficacy estimates.

Insulin dose documentation was available for 9 of 17 patients. Among these, five (55.5%) demonstrated reductions in total daily insulin requirements. Basal insulin was discontinued in two patients (22.2%), and bolus insulin was discontinued in one patient (11.1%). One patient discontinued at least one oral glucose-lowering agent. Changes in diabetes therapy were evaluated descriptively due to incomplete structured dose documentation and heterogeneity in regimens.

### Weight change

Paired baseline and follow-up weight measurements were available for 16 patients. Baseline descriptive weight statistics presented in **Table 1** reflect the full cohort (N = 17), whereas change analyses were performed among patients with paired measurements (n = 16). Mean body weight decreased from 98.64 ± 23.28 kg at baseline to 86.02 ± 19.47 kg at follow-up, corresponding to a mean reduction of −12.63 ± 24.03 kg. Using the Wilcoxon signed-rank test, this change did not reach statistical significance (p = 0.074). The estimated mean difference based on bootstrap resampling was −12.63 kg (95% CI −25.33 to −2.65).

### Safety outcomes

Safety outcomes were assessed throughout follow-up. AKI was evaluated in the seven patients who were not receiving dialysis at baseline. Four of these patients (57.1%) experienced at least one AKI episode, and all four experienced recurrent episodes. AKI percentages are calculated among non-dialysis patients (n = 7). No formal causality assessment between semaglutide exposure and AKI events was performed.

Severe hypoglycemia requiring emergency department evaluation or hospitalization occurred in two patients (11.8%).

Four patients discontinued semaglutide due to adverse events: one due to pancreatitis (5.9%) and three due to gallstones (17.6%). Safety outcomes are summarized in **Table 4**.

**Table 4:** Efficacy and safety outcomes following GLP-1 receptor agonist initiation (N = 17).

Outcome	Result	P-value
Primary efficacy outcomes		
Baseline HbA1c, % (mean ± SD)	9.06 ± 1.69	
Most recent HbA1c, % (mean ± SD)	8.75 ± 2.48	
Change in HbA1c, % (mean ± SD)	−0.31 ± 2.57	0.630
HbA1c change, % (95% CI)*	−0.31 (−1.46 to 0.91)	
Changes in diabetes management		
Total insulin dose decreased, n (%)†	5 (55.5)	
Bolus insulin discontinued, n (%)†	1 (11.1)	
Basal insulin discontinued, n (%)†	2 (22.2)	
≥1 oral hypoglycemic agent discontinued, n (%)	1 (11.1)	
Secondary efficacy outcome		
Baseline body weight, kg (mean ± SD)‡	98.64 ± 23.28	
Most recent body weight, kg (mean ± SD)‡	86.02 ± 19.47	
Change in body weight, kg (mean ± SD)‡	−12.63 ± 24.03	0.074
Weight change, kg (95% CI)*‡	−12.63 (−25.33 to −2.65)	
Safety outcomes		
AKI (non-dialysis patients only), n (%)§	4 (57.1)	
Recurrent AKI (non-dialysis patients), n (%)§	4 (57.1)	
Severe hypoglycemia requiring medical attention, n (%)	2 (11.8)	
Discontinuation due to pancreatitis, n (%)	1 (5.9)	
Discontinuation due to gallstones, n (%)	3 (17.6)	
Cardiovascular outcomes		
Expanded MACE¶, n	3	
Expanded MACE incidence rate (per 100 person-years)	7.69	
Cardiovascular death, n (%)	0 (0.0)	
Nonfatal myocardial infarction, n (%)	0 (0.0)	
Nonfatal ischemic stroke, n (%)	0 (0.0)	
Hospitalization for heart failure, n (%)	3 (17.6)	
Hospitalization for unstable angina, n (%)	1 (5.9)	
Coronary revascularization, n (%)	0 (0.0)	
Mortality outcomes		
All-cause mortality, n (%)	4 (23.5)	
Mortality incidence rate (per 100 person-years)	10.26	

*95% confidence intervals estimated using bootstrap resampling.

†Percentage calculated among patients with available insulin documentation (n = 9).

‡Weight analyses performed among patients with paired baseline and follow-up measurements (n = 16).

§AKI percentages calculated among non-dialysis patients (n = 7).

¶Expanded MACE defined as a composite of cardiovascular death, nonfatal myocardial infarction, nonfatal ischemic stroke, and hospitalization for heart failure. Hospitalization for unstable angina and coronary revascularization were analyzed separately and were not included in the composite endpoint.

Percentages are calculated using the full study cohort (N = 17) unless otherwise specified.

### Cardiovascular outcomes

During accumulated follow-up time totaling approximately 39.0 person-years, three expanded MACE events were observed, yielding an incidence rate of 7.69 events per 100 person-years. Expanded MACE was defined as a composite of cardiovascular death, nonfatal myocardial infarction, nonfatal ischemic stroke, and hospitalization for heart failure.

No cardiovascular deaths, nonfatal myocardial infarctions, or nonfatal ischemic strokes occurred during follow-up. All three expanded MACE events consisted of hospitalizations for heart failure, each occurring in a different patient. No patient experienced more than one component of the composite endpoint.

Hospitalization for unstable angina and coronary revascularization were analyzed separately and were not included in the composite endpoint. One hospitalization for unstable angina occurred, and no revascularization procedures were recorded.

### All-cause mortality

During 39.0 person-years of follow-up, four deaths occurred, corresponding to an incidence rate of 10.26 deaths per 100 person-years. All-cause mortality was analyzed separately from cardiovascular outcomes. Cause-specific mortality could not be independently adjudicated, and deaths are reported descriptively. Precise timing of death relative to semaglutide initiation could not be systematically determined from the structured dataset, as specific dates of death were not consistently captured. No deaths were documented as directly attributable to acute myocardial infarction or stroke. Efficacy and safety outcomes are summarized in **Table 4**.

## Discussion

This study adds to the limited available data on the use of semaglutide in patients with advanced CKD and provides insight over a prolonged follow-up period exceeding three years (median 1,187 days [IQR 602–1,442]), with the majority of patients receiving maintenance hemodialysis (58.8%). The small sample size reflects our deliberate restriction to individuals with ESRD (eGFR <15 mL/min/1.73 m²), a population consistently underrepresented in landmark clinical trials. The American Diabetes Association (ADA) 2026 guidelines recommend semaglutide in patients with advanced CKD without specifying eGFR cutoffs and note that its glucose-lowering effect is preserved at low eGFR levels, in contrast to SGLT2 inhibitors, whose glycemic efficacy diminishes with declining kidney function (30). Similarly, the KDIGO 2022 guidelines recommend semaglutide without defining specific eGFR thresholds (31). In this context, our study reflects real-world prescribing practices in which semaglutide was initiated despite uncertainty regarding optimal eGFR thresholds.

In our cohort, semaglutide use was associated with a modest reduction in HbA1c (−0.31 ± 2.57%, p = 0.630) and a numerically large but statistically non-significant reduction in body weight (−12.63 ± 24.03 kg, p = 0.074). However, the reduction in HbA1c was small and did not reach statistical significance; therefore, it should be interpreted cautiously. Given the heterogeneous follow-up duration and absence of standardized assessment windows, glycemic findings should be viewed as descriptive longitudinal observations rather than definitive efficacy estimates. Reductions in insulin requirements were observed among the nine patients with available dosing documentation; however, because insulin data were incomplete and available for only a subset of the cohort, these findings should be considered exploratory and not generalizable.

The magnitude of weight reduction observed in this cohort was numerically substantial. However, body weight measurements were derived from routine clinical documentation and were not standardized in relation to dialysis sessions. Because pre- and post-dialysis weights can differ significantly due to intradialytic fluid removal and volume shifts, variability in measured weight change is likely, particularly among dialysis-dependent patients. As a result, the observed reduction should be interpreted cautiously. The magnitude of weight change cannot be assumed to represent a precise estimate of adiposity loss and may partially reflect fluid shifts rather than true changes in body composition.

Our findings are directly consistent with prior reports in advanced CKD and ESRD populations. Thomas et al. reported HbA1c reductions of −0.5% (± 1.4) and body weight reductions of −5 kg (± 8) among patients with eGFR <15 mL/min/1.73 m² treated with semaglutide (32). Long et al. demonstrated HbA1c reductions of −0.61% (−1.7, 0.54) and weight reductions of −3.3 kg (−9.0, 2.4) in patients with CKD stage 5 and those receiving dialysis (33). In patients with ESRD on hemodialysis, dulaglutide has been shown to significantly reduce insulin requirements without increasing hypoglycemia (21), and liraglutide improved glycemic control in a placebo-controlled study of dialysis patients (18). Case reports have also described meaningful weight reduction with semaglutide in dialysis-dependent patients (20). Collectively, these findings suggest that the metabolic effects of GLP-1 receptor agonists may be preserved despite advanced renal dysfunction, although robust randomized data in ESRD remain lacking.

With respect to cardiovascular outcomes, three expanded MACE were observed during follow-up, all of which consisted of hospitalizations for heart failure. No cardiovascular deaths, nonfatal myocardial infarctions, or nonfatal ischemic strokes occurred. Expanded MACE in this study was defined as a composite of cardiovascular death, nonfatal myocardial infarction, nonfatal ischemic stroke, and hospitalization for heart failure, while unstable angina and coronary revascularization were analyzed separately and were not included in the composite endpoint. All-cause mortality occurred in four patients and was reported independently.

These findings should not be interpreted as evidence of cardiovascular safety or neutrality. Patients with ESRD have a markedly elevated baseline risk of heart failure hospitalization and mortality independent of glucose-lowering therapy, and the observed event rates likely reflect the substantial comorbidity burden inherent to this high-risk population. Given the small sample size, absence of a comparator group, lack of formal event adjudication, and limited statistical power, no conclusions regarding cardiovascular benefit or harm can be drawn. Although GLP-1 receptor agonists have demonstrated reductions in MACE in large cardiovascular outcome trials among broader T2DM populations (34), direct comparisons to the present cohort are not appropriate, particularly given the exclusion of dialysis-dependent patients from most landmark trials.

All-cause mortality occurred in four patients during follow-up. Cause-specific mortality could not be independently adjudicated, and the precise timing of death relative to semaglutide initiation could not be systematically determined due to the absence of consistently recorded death dates in the structured dataset. Therefore, mortality findings are reported descriptively and should not be interpreted as evidence of treatment effect. Observational data have suggested lower all-cause mortality with GLP-1 RA use compared with other glucose-lowering therapies in advanced CKD (35); however, causal inference cannot be drawn from our data.

AKI occurred in four of the seven non-dialysis patients (57.1%). This proportion appears high; however, it is based on a small denominator and should be interpreted cautiously. Patients with ESRD and advanced CKD are inherently at high risk for AKI due to baseline renal vulnerability, intercurrent illness, volume shifts, and concomitant medications. AKI was identified using creatinine-based KDIGO criteria without systematic adjudication of precipitating factors. Therefore, causality cannot be attributed to semaglutide. Prior studies have reported variable AKI rates with GLP-1 receptor agonists (32), and pre-existing CKD substantially increases AKI risk (36). Severe hypoglycemia occurred in two patients (11.8%), consistent with the elevated hypoglycemia risk observed in advanced CKD populations (37).

This study has several strengths. It directly addresses a critical evidence gap by focusing on patients with ESRD, a population excluded from most GLP-1 RA outcome trials. The prolonged follow-up enhances understanding of longer-term real-world use, and inclusion of both dialysis-dependent and non-dialysis-dependent patients increases pragmatic relevance.

Several limitations should be acknowledged. The retrospective design introduces the potential for information bias, incomplete documentation, and missing data inherent to electronic health record–based research. Detailed prescription-level semaglutide exposure data were unavailable for three patients, limiting complete dose-level characterization and potentially leading to exposure misclassification.

The small sample size and absence of a comparator group substantially limit causal inference and reduce statistical power to detect infrequent adverse events or draw definitive conclusions regarding cardiovascular outcomes. The expanded MACE definition included hospitalization for heart failure, a common event in ESRD, and therefore cardiovascular findings should be interpreted descriptively rather than as evidence of benefit or harm.

Follow-up windows were not prespecified, and HbA1c and weight reflect the most recent available clinical measurements with heterogeneous follow-up duration. Accordingly, these outcomes represent longitudinal real-world observations rather than standardized timepoint efficacy estimates. The HbA1c reduction was modest and not statistically significant. Insulin dose data were available for only nine patients and were analyzed descriptively.

Body weight measurements were not standardized relative to dialysis sessions; pre- and post-dialysis fluid shifts may have influenced the magnitude of observed weight change.

Safety events were identified through clinical documentation without independent adjudication. Pancreatitis and gallstones were based on recorded diagnoses without systematic laboratory or imaging confirmation, and gastrointestinal adverse events were not predefined unless leading to discontinuation. AKI was defined using creatinine-based KDIGO criteria and evaluated among seven non-dialysis patients; the apparent high incidence should be interpreted cautiously given the small denominator and high baseline AKI risk in advanced CKD. Episode-level staging and precipitating factors could not be systematically verified.

All-cause mortality was reported descriptively, and cause-specific mortality and precise timing relative to cardiovascular events could not be independently adjudicated. Treatment duration was estimated using prescription span, and adherence could not be confirmed.

Accordingly, these findings should be interpreted as hypothesis-generating. Larger, prospective, controlled studies with standardized outcome adjudication and predefined follow-up intervals are needed to better define the safety profile, optimal dosing strategies, and clinical impact of semaglutide in patients with ESRD.

## Conclusion

In this small retrospective cohort of patients with ESRD, semaglutide use was associated with descriptive changes in glycemic parameters, body weight, and clinical outcomes over prolonged follow-up. However, the observed HbA1c reduction was modest and not statistically significant, and safety events, including heart failure hospitalization, AKI, and mortality, reflect the high baseline risk of this population. Given the small sample size, absence of a comparator group, heterogeneous follow-up, and limitations inherent to retrospective data collection, no conclusions regarding glycemic efficacy, cardiovascular safety, or overall safety profile can be drawn. These findings should be considered exploratory and hypothesis-generating. Larger, prospective, controlled studies with standardized outcome adjudication are required to better define the safety and clinical impact of semaglutide in patients with ESRD.

## Data Availability

The data supporting the findings of this study are not publicly available due to ethical and legal restrictions related to patient privacy and institutional regulations. De-identified data may be made available from the corresponding author upon reasonable request, subject to approval by the Institutional Review Board and the participating institutions. Requests to access the datasets should be directed to Lama Alfehaid, Fehaidl@ksau-hs.edu.sa.
